# Computational Fluid Dynamics Simulations of Contrast Agent Bolus Dispersion in a Coronary Bifurcation: Impact on MRI-Based Quantification of Myocardial Perfusion

**DOI:** 10.1155/2013/513187

**Published:** 2013-02-28

**Authors:** Regine Schmidt, Dirk Graafen, Stefan Weber, Laura M. Schreiber

**Affiliations:** Section of Medical Physics, Department of Radiology, University Medical Center of the Johannes Gutenberg University, 55131 Mainz, Germany

## Abstract

Contrast-enhanced first-pass magnetic resonance imaging (MRI) in combination with a tracer kinetic model, for example, MMID4, can be used to determine myocardial blood flow (MBF) and myocardial perfusion reserve (MPR). Typically, the arterial input function (AIF) required for this methodology is estimated from the left ventricle (LV). Dispersion of the contrast agent bolus might occur between the LV and the myocardial tissue. Negligence of bolus dispersion could cause an error in MBF determination. The aim of this study was to investigate the influence of bolus dispersion in a simplified coronary bifurcation geometry including one healthy and one stenotic branch on the quantification of MBF and MPR. Computational fluid dynamics (CFD) simulations were combined with MMID4. Different inlet boundary conditions describing pulsatile and constant flows for rest and hyperemia and differing outflow conditions have been investigated. In the bifurcation region, the increase of the dispersion was smaller than inside the straight vessels. A systematic underestimation of MBF values up to −16.1% for pulsatile flow and an overestimation of MPR up to 7.5% were found. It was shown that, under the conditions considered in this study, bolus dispersion can significantly influence the results of quantitative myocardial MR-perfusion measurements.

## 1. Introduction

Coronary artery disease (CAD) is one of the main causes of death in industrial nations. It usually becomes manifested in coronary artery stenosis of varying severity. A common consequence of CAD is a reduced perfusion of the myocardium [1], which may lead to ischemic heart disease and, possibly, cardiac infarction. Therefore, evaluation of myocardial blood flow (MBF) is an important approach to determine regional perfusion deficits. Ideally, this measurement should be performed under physical rest and pharmacologically induced hyperemia, since arterial stenosis results in more severe perfusion deficits at hyperemia. The myocardial perfusion reserve (MPR) is defined as the ratio of the MBF at hyperemia and at rest. One established method for detection of the MBF is magnetic resonance imaging (MRI), which has the advantage of being noninvasive and which does not make use of ionizing radiation. By using T1-weighted contrast-enhanced first-pass MRI, myocardial perfusion can be assessed qualitatively [2], semiquantitatively [3, 4], and quantitatively [5–7]. Quantitative determination of MBF from MRI data can be performed with the help of tracer kinetic models like the multiple path, multiple tracer, indicator dilution, 4 region model (MMID4) (National Simulation Resource, University of Washington, Seattle, USA) [8]. This requires the information of how the contrast agent arrives at the tissue of interest, that is, the arterial input function (AIF). Ideally, this AIF should be measured in a vessel supplying the tissue of interest for any kind of perfusion measurement. However, this is not feasible in image-based myocardial perfusion analysis. 

In practice, the AIF is usually estimated from the blood pool of the left ventricle (LV). Unfortunately, dispersion of the contrast agent bolus might occur between the LV and the myocardium. Dispersion corresponds to a deformation of the original contrast agent concentration-time curve. Depending on the cross-sectional location and the phase in the pulsatile pattern, the contrast agent particles travel with different velocities. This leads to a stretch of the contrast agent bolus in time and space, which results in a prolonged bolus with a reduced maximal contrast agent concentration, even at laminar flow profiles. Negligence of this dispersion can lead to an error in quantification of the MBF and MPR. Using a single exponential shaped transfer function, a previous study by Schmitt et al. has shown that depending on the amount of dispersion a systematic underestimation of blood flow arises [9].

Recently, several studies based on computational fluid dynamics (CFD) investigated blood flow in coronary arteries. Most of them examined the correlation between wall shear stress and atherosclerosis, for example, [10–13]. Calamante et al. performed CFD simulations to estimate the dispersion of a contrast agent bolus in cerebral vessels [14]. Previous CFD simulations examining the bolus dispersion in coronary vessels performed by Graafen et al. used an idealized single vessel geometry considering both constant [15] and pulsatile [16] flows. Both demonstrated an underestimation of the MBF and an overestimation of the MPR if bolus dispersion is neglected. In the worst case, this can result in a wrong classification of an ill patient as healthy, that is, a false negative finding. However, the amount of the underestimation of the MBF was negligible when compared with a typical error of interquartile range of about ±20% for myocardial perfusion MRI in healthy volunteers [7].

The aim of this study was to expand this work from a single vessel geometry to a more realistic geometry, that is, an idealized bifurcation of the left coronary artery where a stenosis is located in one of the branches. Results from different inlet conditions, pulsatile and constant flow, physical rest and hyperemia, and different outflow conditions for the two branches are compared and discussed. Afterwards, the influence of bolus dispersion on MBF and MPR values under these conditions is determined.

## 2. Materials and Methods

### 2.1. Geometry

**Figure 1:**
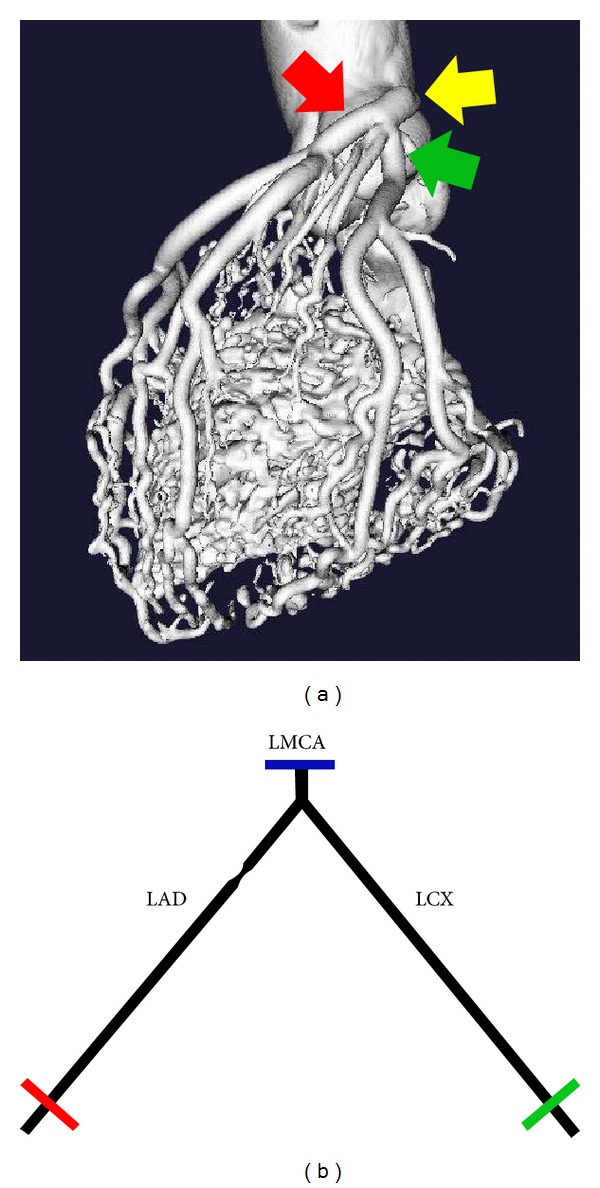
(a) 3D volume rendered CT data set obtained from a corrosion preparation of a human heart showing the anatomical positions of the LMCA (yellow arrow), LAD (red arrow), and LCX (green arrow). (b) Top view of the bifurcation geometry. Cross sections shown in Figure 5 are marked in the respective colors.

An idealized geometry of the bifurcation of the left main coronary artery (LMCA) to the left anterior descending (LAD) and the left circumflex (LCX) was generated based on the literature data of human coronary artery dimensions (cf.  Figure 1). This special bifurcation was investigated in this study since it is the main bifurcation of the left coronary artery which typically supplies the main fraction of the heart muscle with blood and is a frequent location of stenosis. The advantage of studying an idealized rather than a real vessel geometry is that influencing factors can be varied more easily and controlled.

 The geometry was generated using the commercial software package Gambit (Gambit 2, Fluent GmbH, ANSYS, Darmstadt, Germany). Each branch was considered to be cylindrical. The radius of the LMCA was chosen to be 2.25 mm and the radius of LAD and LCX to be 1.78 mm each, which corresponds to an average value of the literature data for normal RCA dominant male [17]. The angle between LAD and LCX was set to 80° [18]. The length of the arteries was chosen to be 10 mm centerline length inside the LMCA and 100 mm centerline length inside the LAD and LCX. To minimize possible influences caused by the outlet boundary conditions, the geometry was extended 10 mm behind the outlets. The results obtained in this region were neglected in the subsequent analysis. A symmetrical cosinusoidal shaped stenosis with a length of 10 mm and an area reduction of 80% were included at the LAD. The center of this stenosis was located at a distance of 25 mm behind the bifurcation which was estimated with respect to the literature data concerning typical locations of stenosis [19].

In our model, the cross-sectional area increases by a factor of 1.25 at the bifurcation, which lies within the range reported in the literature [20]. This fact is an important issue since the flow rate stays constant inside the arterial tree if the assumption of rigid vessel walls is made. Therefore, an increase in area corresponds to a decrease in blood velocity, whereas the average velocity is an important factor influencing the dispersion of the contrast agent [16].

**Figure 2:**
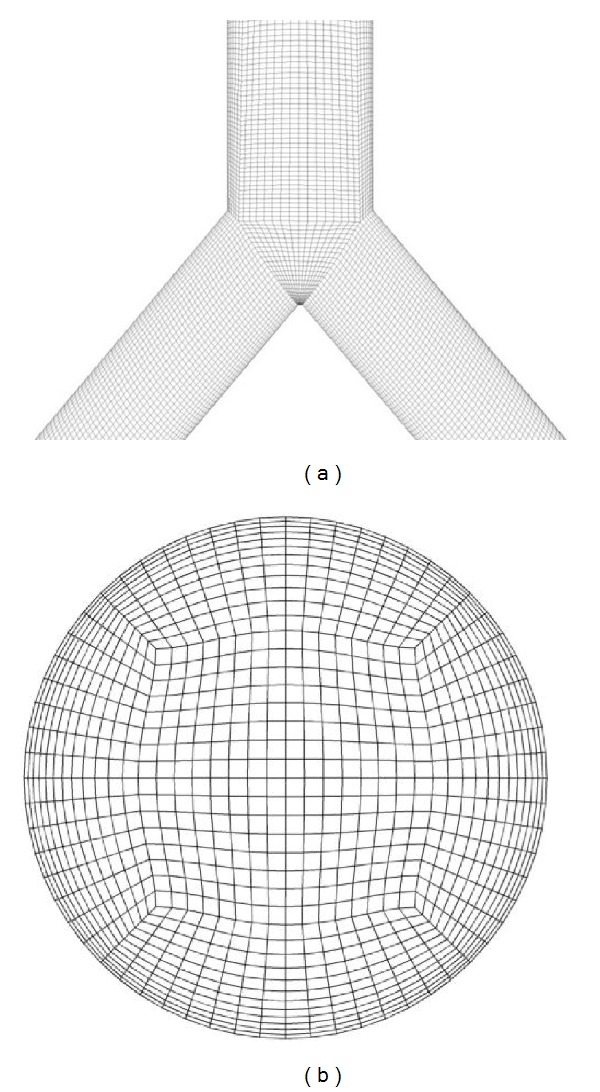
(a) Mesh in the bifurcation region. (b) Hexahedral O-grid type mesh of the vessel inlet.

The vessel geometry was meshed using the commercial software package ICEM CFD (ICEM CFD 12, ANSYS, Darmstadt, Germany). A hexahedral, O-grid type mesh was created for discretizing the fluid domain to achieve a sufficient alignment of the grid with the predominant flow direction (cf.  Figure 2(a)). The O-grid type mesh showed a refined boundary layer to account for the large velocity gradient located close to the wall of the domain (cf.  Figure 2(b)). Furthermore, a refinement of the grid in flow direction was performed inside the stenosis region to avoid strongly deformed stretched elements and to guarantee a better spatial resolution in this area of strong flow changes. For the latter reason, the grid was refined close to the bifurcation area as well. At the grid refinement procedure, the length of the elements in flow direction was linearly reduced up to a factor of 3 inside the stenosis region, and up to a factor of 1.3 inside the bifurcation region. To improve the accuracy and stability of the solution, several mesh variations were performed to maximize the grid quality. Parameters describing the quality of a hexahedral grid were investigated and improved. These are determinants, angles, and warpages of the grid elements [21]. The final grid exhibited a good overall quality with a minimal determinant of 0.5 (recommended: >0.3 [21]), a minimal angle of 35 degrees (recommended: >18 degrees), and a maximal warpage of 14 (ranges from 0 (low distortion) to 90 (high distortion)). The grid consisted of 1.38 million hexahedral elements. 

### 2.2. Boundary Conditions

As inlet boundary condition a pulsatile velocity pattern was assigned to the inlet surface. The pattern was gained from data points of a phase-contrast MRI measurement inside the LMCA by Schiemann et al. [22]. A polynomial function of the 18th order was fitted to the data to achieve a smoother curve and to improve temporal interpolation of the data. The resulting pattern corresponds to a typical heart rate of 60 beats per minute.

 The performed simulations can be divided into two sets of simulations (“full autoregulation” and “limited autoregulation”). Each set consists of four simulations: pulsatile and constant flows for physical rest and hyperemia. Physical rest corresponds to an unexcited physical state with normal heart rate. In contrast, hyperemia is usually correlated with increased heart rate and increased blood velocity and, therefore, increased blood flow. Since perfusion deficits show more effect at hyperemia, myocardial blood flow should be measured at hyperemia for authentic results. Different outflow conditions were considered for each set of simulations, and the original velocity pattern was scaled to account for the two physical states (rest and hyperemia) and several differing flow conditions through the LAD and the LCX, respectively. The values of the different outflow conditions, scaling factors, and constant velocities for the two sets of simulations are summed up in Table 1. Both sets of simulations are explained more detailed in the following.

**Figure 3:**
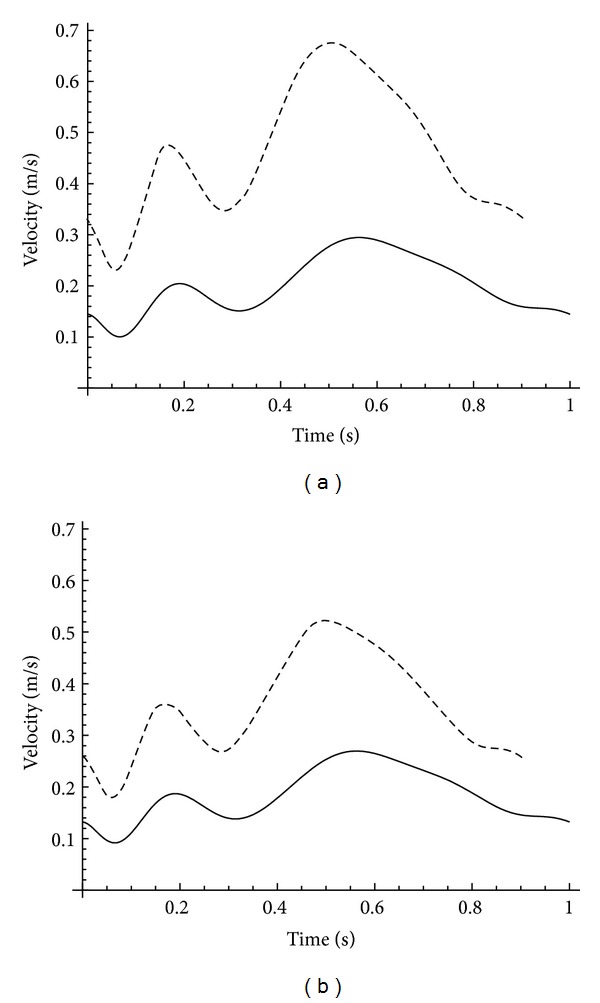
Processed velocity pattern measured inside the LMCA (a) for full autoregulation for rest (solid) and hyperemia (dashed) and (b) for limited autoregulation for rest (solid) and hyperemia (dashed). The heart rate is increased by 10% for hyperemia.

At the first set of simulations an outflow condition of 50% both through the LAD and LCX branches was fixed. This simulates full autoregulation of the pressure drop across the stenosis by vasodilation of the downstream vessels [23], that is, a condition where vasodilation results in a decrease of resistance in these downstream vessels [24]. For simulation of hyperemia the pattern has been scaled up by the factor of 2.3 [25, 26], which corresponds to an MPR of 2.3. The heart frequency increases by about 10–15% during adenosine-induced hyperemia [27–29]. Thus, the duration of the cardiac cycle was reduced in our simulations by 10% to 0.9 s in this case, which corresponds to an increase in heart rate to 66.67 beats per minute, respectively.  Figure 3(a) shows the velocity patterns used for the pulsatile condition of this set of simulations. For the simulations performed under constant velocity conditions the time-averaged velocity of the pulsatile pattern has been used as inlet velocity. This resulted in values of 0.200 m/s for rest and 0.460 m/s for hyperemia, respectively. This set of simulations was characterized as “full autoregulation” in the rest of this paper.

More realistically, the stenosis may affect the blood flow velocity even at the vessel branch inlet. That means the influence of stenosis is not fully compensated by the downstream vessels which corresponds to limited autoregulation. A study performed by Segal et al. showed a reduced velocity proximal and distal to stenosis at measuring the mean time-averaged peak velocity before and after angioplasty using a Doppler guide wire [26]. Therefore, the simulations were repeated using adapted outflow conditions considering reduced flow through the stenosed LAD. According to the results of Segal et al., the velocity in the stenosed LAD was reduced by a factor of 0.829 proximal to the stenosis. To retain average velocity in the healthy vessel and to achieve a velocity reduced by a factor of 0.829 in the stenosed LAD, the velocity pattern was scaled, respectively, and the outflow was varied to 45.3% for LAD and 54.7% for LCX. For the case of resting condition, the pattern shown in Figure 3(a) was scaled by a factor of 0.915. 

Furthermore, Segal et al. measured the coronary flow reserve using time-averaged peak velocity for healthy and stenosed coronary arteries [26]. For normal vessels they obtained an average coronary flow reserve ratio of 2.3, for stenosed vessels they determined an average value of 1.5 proximal to the stenosis, respectively. That means the velocity was increased by a factor of 1.5 inside the stenosed vessel and by a factor of 2.3 inside the normal vessel for hyperemic condition. To implement these ratios in our simulations, the outflow was set to 35.1% for LAD and 64.9% for LCX and the original pulsatile rest velocity pattern was scaled by a factor of 0.771 × 2.3 = 1.773. The scaled velocity patterns for rest and hyperemia can be seen in Figure 3(b). For the constant flow condition, the time-averaged velocity of the flow patterns has been used as well, which was 0.183 m/s for rest and 0.355 m/s for hyperemia, respectively. This set of simulation was characterized as “limited autoregulation” in the following.

Plug flow was assumed as inlet condition for the velocity profile at the LMCA. If a smaller vessel diverges almost perpendicular from a larger vessel, there is no fully developed parabolic velocity profile present at the inlet of the smaller vessel. Therefore, we think this is an acceptable approximation for our simulations since the origin of the coronary arteries arises from the ascending aorta.

The mass fraction of the injected contrast agent bolus *Y*_CA_ can be described by a gamma-variate function at the inlet of our geometry [15, 16, 30]

(1) $$\begin{array}{c} Y_{\text{CA}}\left(t,z=0\right)=\{\begin{array}{cc} {a(t-t_{0})}^{b}e^{-c(t-t_{0})} & t>t_{0},\\ 0 & t\leq t_{0}, \end{array} \end{array}$$

where the parameters have been estimated by fitting the AIF measured inside the LV of a volunteer MRI measurement as *a* = 1.013 × 10^−3^, *b* = 2.142, and *c* = 0.454 s^−1^. The parameter *t*_0_ describes the delayed arrival of the bolus. It was set to the end of three cardiac cycles to assure a fully developed periodicity of the flow field before contrast agent arrival. After these three cardiac cycles, a contrast agent transport through the geometry of a duration of 50 s was simulated.

### 2.3. CFD Simulations

The governing equations have been solved using the commercial software package Fluent (Fluent 14, ANSYS, Darmstadt, Germany). CFD simulations were performed at the High Performance Cluster Elwetritsch (Elwetritsch, RHRK, TU Kaiserslautern, Germany) on 8 cores each. Sixteen cores were used for the simulation of pulsatile blood flow at hyperemia for full autoregulation due to the long computing time. 

As pressure-velocity coupling method the “pressure-implicit with splitting of operators” (PISO) algorithm [31] was used. A different time step size from 0.001 s up to 0.010 s was used for the individual simulations to account for rest and hyperemia, constant and pulsatile flow, respectively. The number of time steps was varied to account for the specified duration of the simulation of 53.0 s for rest and 52.7 s for hyperemia, respectively. For the spatial discretization scheme the second-order upwind scheme was set for momentum, and contrast agent, the transient formulation was chosen to be second-order implicit, and the “pressure staggering option” (PRESTO!) scheme [32] was chosen as pressure interpolation scheme. The convergence limit for relative errors was fixed to 1 × 10^−3^. Flow was considered to be laminar and incompressible. No slip boundary condition was assumed at the vessel wall. Vessel walls were assumed to be rigid and motionless.

To simulate the transport of contrast agent, a mixture of contrast agent and blood was assumed. Due to the low concentration of contrast agent in blood (≤0.32 mass%), the rheological properties of the contrast agent were neglected and typical values of blood properties were used instead. Blood was considered to be a Newtonian fluid, and a typical density of *ρ* = 1050 kg/m^3^ and a dynamic viscosity of *η* = 0.004 kg/(m∗s) were chosen. The diffusion coefficient of the contrast agent in blood at body temperature was estimated by Graafen et al. to be *D* = 1.5∗10^−10^ m^2^/s [16]. In this case Gd-DTPA, which is a typical contrast agent used at clinical MRI measurements, was considered. 

### 2.4. Determination of the Variance of the VTF as a Measure of Bolus Dispersion

Mathematically, dispersion of the AIF can be specified by the convolution of the undispersed AIF_LV_, which is usually measured in the LV, and a convolution kernel, which is typically called vascular transport function (VTF) [14–16, 33]

(2) $$\begin{array}{c} {\text{AIF}}_{\text{toi}}=\text{VTF}⊗{\text{AIF}}_{\text{LV}}, \end{array}$$

where AIF_toi_ describes the true dispersed AIF at the tissue of interest. The VTF is a function of time and space along the vessel. The variance *σ* ^2^ of the VTF can be used to characterize the dispersion of the bolus. It can be calculated as [15, 16, 34]

(3) $$\begin{array}{c} \sigma_{\text{VTF}}^{2}\left(z\right)=\frac{\int_{0}^{\infty }{\left[t-\text{MVTT}(z)\right]}^{2}\text{VTF}\left(t,z\right)dt}{\int_{0}^{\infty }\text{VTF}\left(t,z\right)dt}, \end{array}$$

where MVTT corresponds to the mean vascular transit time

(4) $$\begin{array}{c} \text{MVTT}\left(z\right)=\frac{\int_{0}^{\infty }\text{VTF}\left(t,z\right)tdt}{\int_{0}^{\infty }\text{VTF}\left(t,z\right)dt}. \end{array}$$

To avoid deconvolution, the variance of the VTF can be evaluated with the help of the zeroth, first, and second integral moment of the AIFs, where the AIFs correspond to the cross-sectional averages of the mass fraction of contrast agent *Y*_CA_ in this study, [15, 16, 34] by

(5) σVTF2(z)=YCA(2)(z)YCA(0)(z)−YCA(2)(z=0)YCA(0)(z=0)+[YCA(1)(z=0)YCA(0)(z=0)]2−[YCA(1)(z)YCA(0)(z)]2.

This can be calculated from the input and the results of the CFD simulations.

### 2.5. Estimation of MBF and MPR Determination Error

The estimation of the influence of contrast agent bolus dispersion on determination of the MBF was performed in three steps. For this purpose, the simulation software XSIM (National Simulation Resource, University of Washington, Seattle, USA) was used in combination with the MMID4 model. The tracer kinetic model MMID4 enables the simulation of blood-tissue exchange of a contrast agent. It provides ways to explore the delay and dispersion of the considered contrast agent between the location of injection and the target organ. This model can be used to examine the physiology of the exchange process and to analyze experimental data. In this study, MMID4 was used to simulate the development of a contrast agent bolus inside arteries close to the tissue of interest and the microcirculation, which is composed of 20 pathways in this model, and to analyze the obtained data analog to the procedure of MR-perfusion measurements [7]. The macroscopic blood flow pattern and the contrast agent bolus dispersion inside the larger main coronary arteries, that is, the scope of this study, are neglected by MMID4. Each single microcirculation pathway includes a nonexchanging arteriole and a blood tissue exchange unit to simulate the movement of the contrast agent between intravascular and interstitial regions. Distribution of blood flow to the different pathways is characterized by a probability density function. The mean blood flow is equal to the MBF in this case. One data point of each cardiac cycle of the simulation results was used for this procedure. Forty-four cardiac cycles of each mass fraction-time curve were considered, which is a typical value used in clinical MR-perfusion measurements.

**Figure 4:**
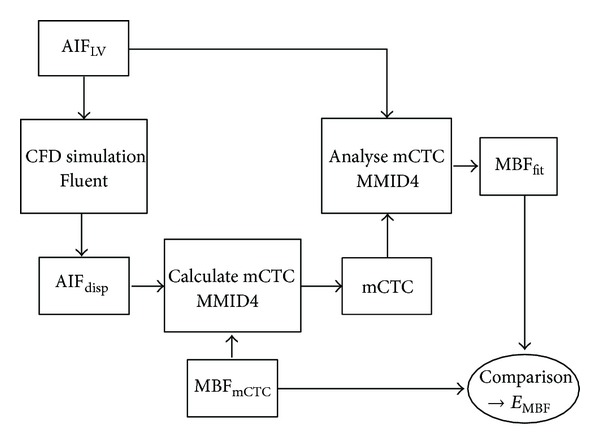
Sketch of the procedure to determine the error in the MBF using Fluent and MMID4. The acronym “mCTC” stands for myocardial concentration-time curve.

 To estimate the error in the MBF determination caused by the negligence of dispersion, a three-step procedure is used, consisting of the following steps (cf.  Figure 4).

1. Generation of the myocardial concentration-time curves for specific MBF and MPR values using the dispersed AIF obtained at the CFD simulations.
2. Determination of the MBF with the AIF_LV_ and the generated myocardial concentration-time curves. This corresponds to the typical clinical procedure of MBF quantification.
3. Comparison of the MBF values assumed for 1 and the values obtained at 2.

In the first step, the dispersed AIF was used to calculate a typical myocardial concentration-time curve. As density function, a slightly right-skewed lagged function [35] with a relative dispersion of RD = 0.55 [36] was employed. Further hemodynamic parameters were set to typical literature values [5–8, 16, 36]: regional dispersion (RD) in all vessels, including arteries, arterioles, venules, and veins = 0.48, volume of arteries (*V*_art_) = 0.02 mL/g, interstitial volume (*V*_isf_) = 0.35 mL/g, volume of the veins (*V*_ven_) = 0.02 mL/g, volume of the venules (*V*_venl_) = 0.03 mL/g, plasma volume of the capillaries (*V*_*p*_) = 0.04 mL/g, permeability surface area (PS) product = 1 mL/(g∗min), delay between inflow in the LV and the myocardium = 0 s. The volume of the arterioles (*V*_artl_) was chosen to be 0.03 mL/g for rest condition and 0.06 mL/g for hyperemia condition to account for dilatation of the arterioles at hyperemia, respectively. Furthermore, MBF was fixed to typical values of 1 mL/(g∗min) for rest and 2.3 mL/(g∗min) and 1.5 mL/(g∗min) for hyperemia, respectively.

In the second step, the myocardial concentration-time curve calculated in the previous step was used in combination with the original undispersed AIF obtained inside the LV to quantify the MBF analog to the procedure of MR-perfusion measurements. The fitting algorithm SENSOP [37] of the XSIM software, which is an implementation of the Levenberg-Marquardt algorithm, was used for this purpose. For the fitting procedure, four parameters were considered to be free within certain boundaries [16]: MBF (limits: 0 and 7 mL/(g∗min)), permeability surface area (PS) product (limits: 0.25 and 8.00 mL/(min∗g)), delay between inflow of the contrast agent in the LV and the myocardium (limits: −1 and 3 s), and the plasma volume of the capillaries (*V*_*p*_) (limits: 0.03 and 0.09 mL/g). All other parameters were kept constant. This approach is similar to that used in typical clinical studies. The initial values of the four free parameters were chosen to be similar to the values used for the creation of the concentration-time curve at the first step.

In the third step, the error in the MBF that arises due to the application of the undispersed AIF is described as the relative deviation of the MBF values of both previous steps [16]

(6) $$\begin{array}{c} E_{\text{MBF}}=\frac{{\text{MBF}}_{\text{fit}}-{\text{MBF}}_{\text{mCTC}}}{{\text{MBF}}_{\text{mCTC}}}, \end{array}$$

where MBF_fit_ is the MBF value calculated at the fitting procedure neglecting dispersion, and MBF_mCTC_ is a typical MBF value, which was set for the generation of the myocardial concentration-time curve.

The MPR values were set for the single simulations as described in the Section 2.2. The error in MPR was calculated analogous to (6) as

(7) $$\begin{array}{c} E_{\text{MPR}}=\frac{{\text{MPR}}_{\text{fit}}-{\text{MPR}}_{\text{mCTC}}}{{\text{MPR}}_{\text{mCTC}}}. \end{array}$$

## 3. Results

**Figure 5:**
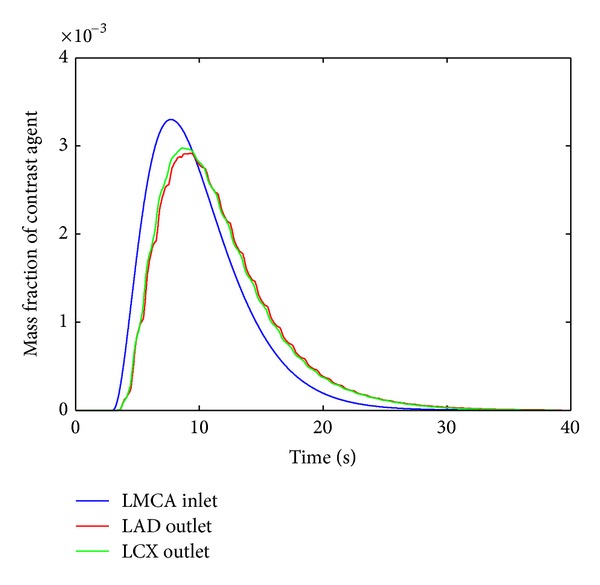
Mass fraction of the contrast agent as a function of time for pulsatile flow under resting condition for limited autoregulation. The positions of the respective cross sections are marked in Figure 1(b). The small oscillations of the contrast agent mass fraction at the outlets are caused by the pulsatile flow.

The mass fraction of the contrast agent was recorded at several cross-sectional positions along the vessel geometry (cf.  Figure 5). They were arranged perpendicular to the centerlines with an intersection distance of 2.5 mm.  Figure 5 illustrates that the shape of the bolus is clearly distorted because of bolus dispersion. Graphs look similar for hyperemia and full autoregulation. Differences between the conditions will be examined next.

### 3.1. The Variance of the VTF as a Measure of Bolus Dispersion

**Figure 6:**
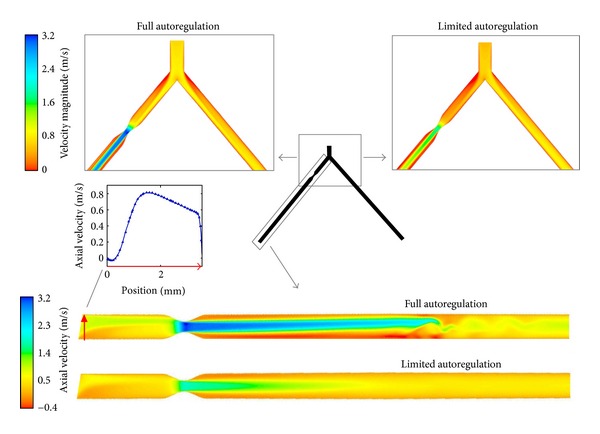
Top: velocity magnitude in the bifurcation region for hyperemia at the time step of maximum velocity for pulsatile flow. Bottom: axial velocity inside the stenotic LAD for the case of hyperemia at the time step of maximum velocity for pulsatile flow. The LAD branches are scaled by a factor of 1.5 in radial direction for better visualization. Axial velocity profile along a radial line (red arrow) directly behind the bifurcation is presented (middle, left).

Representative results of the velocity distribution inside the geometry are shown in Figure 6. In the region closely behind the bifurcation, velocities are strongly reduced at the outer vessel walls, and even small negative axial velocities are found. In contrast, flow is accelerated close to the inner walls, resulting in a skewed velocity profile up to the beginning of the stenosis. Therefore, no parabolic velocity profile is formed in this region. At the beginning of the stenosis, the axial velocity profile is broadened compared to a laminar parabolic velocity profile. Behind the center of the stenosis a jet-like flow develops in the middle of the vessel. Moreover, a recirculation zone with small negative axial velocities develops close to the walls directly behind the stenosis. For the case of full autoregulation shown in Figure 6, the formation of an eddy is observed about 60 mm behind the stenosis.

**Figure 7:**
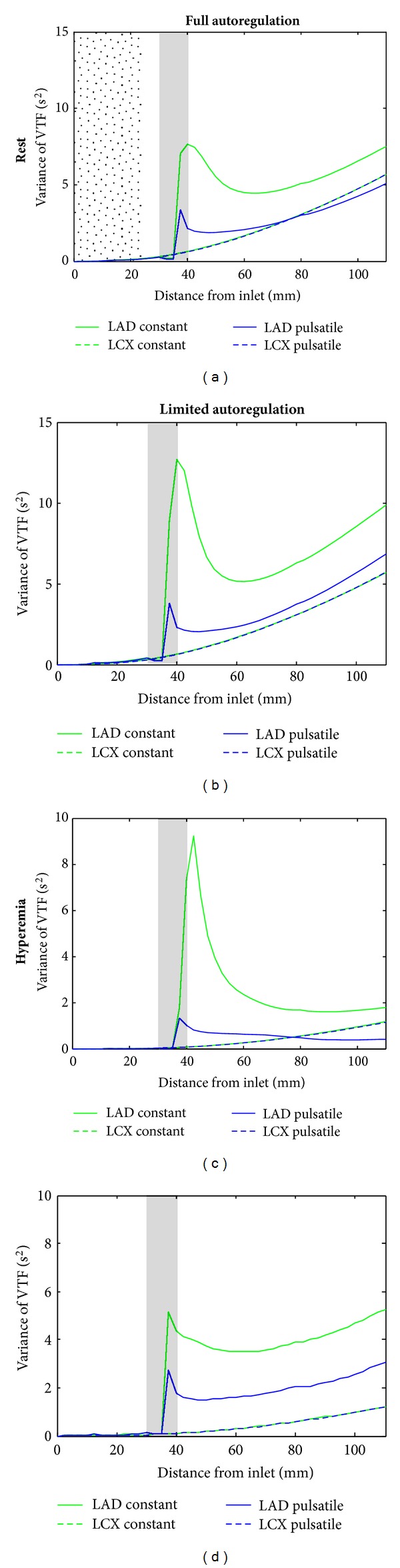
The variance of the VTF as a function of the distance from the inlet. Results of the stenotic LAD are represented as solid line and results of the normal LCX as dashed line. The line representing variance inside the healthy LCX for constant flow is covered by the corresponding line for pulsatile flow. The position of the stenosis is highlighted in grey. The dotted range marked in (a) is shown in more detail in Figure 8.

**Figure 8:**
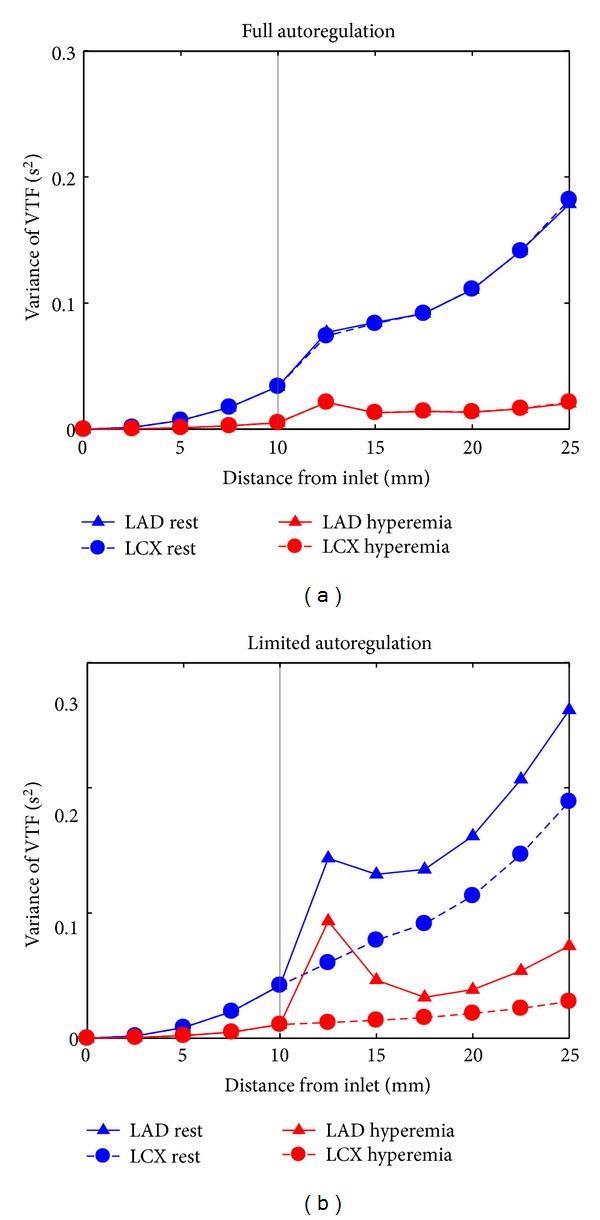
The variance of the VTF as function of the distance from the inlet for the bifurcation region for pulsatile flow for (a) full autoregulation and (b) limited autoregulation. The dotted range in Figure 7(a) is shown here in more detail. The data representing the variance inside the LCX and the corresponding data representing the LAD are partly covering each other. The position of the bifurcation, that is, the last point of the single LMCA centerline, is highlighted in grey.

The variance of the VTF along the LAD and LCX branches for all conditions considered in this study is shown in Figure 7. Results of the dispersion analysis in the bifurcation region are shown in Figure 8. Directly behind the bifurcation an initial increase of the dispersion is observed except in the unconstricted LCX branch in the case of limited autoregulation. The increase of the VTF variance is slightly reduced within a range of about 10 mm in the region close behind the bifurcation. This trend can be explained by the deformation and displacement of the velocity profile in this region.

Dispersion in the stenotic vessel exceeds that in the normal vessel, except for the cases of pulsatile flow for full autoregulation. In the region of the stenosis, the dispersion is decreasing at first. After passing the center of the stenosis, a rapid increase of the dispersion can be seen. Downstream the end of the stenosis, the dispersion is slowly approximating the trend of the dispersion inside the unconstricted vessel.

The general trend of the variance resembles each other for pulsatile and constant flow inside the stenotic branch. However, the leap after passing the center of the stenosis is considerably smaller for pulsatile flow. Furthermore, the dispersion stays smaller inside the constricted vessel for pulsatile flow in comparison to constant flow up to the end of the vessel section. In the normal branch, the variance of the VTF is almost equal for constant and pulsatile flow, respectively. The relative difference at the outlet is less than 3%.

A significantly larger dispersion was found in the stenotic branch for the case of limited autoregulation as compared to full autoregulation. This result might be explained by the reduced flow and, therefore, reduced velocity through the stenotic branch for the case of limited autoregulation [15, 16]. Since the outflow condition was chosen to keep a constant mean flow in the normal branch for both sets of simulations, the change in dispersion in this branch is marginal with a maximal relative deviation of 4.5% at the outlet.

### 3.2. Influence of Bolus Dispersion on MBF and MPR Quantification

**Figure 9:**
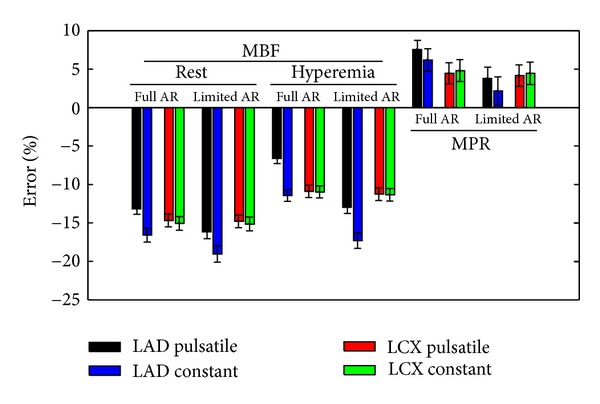
Errors in the MBF and MPR if bolus dispersion is neglected. The acronym “AR” stands for autoregulation.

The deviations of the MBF and the MPR values due to bolus dispersion are illustrated in Figure 9. In general, a systematic underestimation of MBF is observed. It ranges between −11.0% and −19.1% for constant flow, and between −6.6% and −16.1% for pulsatile flow. A clear difference can be seen in the error of MBF for pulsatile flow and constant flow inside the stenotic LAD. Since the underestimation of the MBF is larger for resting condition than for hyperemia, an overestimation of the MPR in the range of 2.2% to 6.2% for constant flow and 3.8% to 7.5% for pulsatile flow can be observed. For the case of fully autoregulated flow, the MPR overestimation is larger in the stenotic LAD branch in comparison to the normal LCX branch, whereas it was found to be smaller for the case of limited autoregulation.

## 4. Discussion

In this study, the bolus dispersion inside a simplified coronary bifurcation geometry was investigated using CFD simulations. Even though the increase in dispersion was found to be reduced within the bifurcation region, a not negligible underestimation in MBF due to bolus dispersion was observed. A systematic underestimation of the MBF up to −16.1% for pulsatile flow and an overestimation of the MPR up to 7.5% were found.

### 4.1. The Variance of the VTF as a Measure of Bolus Dispersion

#### 4.1.1. Bolus Dispersion inside the Bifurcation Region

Figure 6 nicely explains most of our observations. In general, the increase in dispersion is slightly reduced in the region closely behind the bifurcation. Due to the asymmetric and deformed velocity profile in this region, the velocity profile is not laminar. Moreover, close to the inner walls of the bifurcation region, the axial velocity varies less in radial direction compared to the case of a parabolic velocity profile. This leads to a relatively small dispersion (cf. Figure 8). 

 Previous studies found that the bulk velocity has a high impact on dispersion, showing negative correlation [15, 16]. Therefore, the initial increase in dispersion might be explained by the velocity reduction at the inlet of the branch due to the reduced flow. The effect is most distinctive in the stenotic branch for the case of limited autoregulation which correlates with the strongest velocity reduction. Additionaly, the opposing effect of the small recirculation zone at the outer walls directly behind the bifurcation and the acceleration close to the inner walls might disperse the bolus. This would be a similar but reduced effect as that already observed at the exit of a stenosis inside a single vessel [15, 16]. 

Similar observations regarding the flow inside bifurcations were made in other CFD studies of bifurcations [13, 38, 39]. One important finding was the deformed velocity profile in the bifurcation region, which was skewed towards the inner walls (cf. Figure 6). Furthermore, a reduced velocity at the outer walls was found in this region. In contrast, the velocity profiles Ponzini et al. showed in their study were skewed towards the opposite direction [40]. We think this results from the general curvature of the 3D geometry used there. However, the influence on dispersion should be the same as in our study, since a laminar velocity profile is not formed in all cases and the direction of the deformation should not have much influence on the dispersion.

#### 4.1.2. Bolus Dispersion inside the Stenosis Region

A larger dispersion was found in six out of eight simulations inside the stenotic branch compared to the results for the normal branch (cf. Figure 7). Inside the stenosis region, the variance of the VTF shows the typical behavior as already described by Graafen et al. [15, 16]. The initial decrease in variance at the beginning of the stenosis results from the smaller radial variations of the axial velocity profile at this region. The rapid increase in dispersion behind the center of the stenosis is a consequence of the formation of a jet-like stream in the center of the vessel and the recirculation zone close to the wall. Due to these two opposedly contributing effects, the contrast agent bolus is strongly dispersed in this region. Afterwards, the variance is slowly adapting to the trend of the unconstricted vessel again because of the formation of a parabolic velocity profile. However, in contrast to most of our results, Graafen et al. found the dispersion to be smaller inside the stenotic vessel compared to the results for an unconstricted vessel at the end of the considered section [16]. Since the LAD stenosis is placed 25 mm behind the bifurcation in our study, the deformed and displaced velocity profile affects the dispersion inside the stenosis region as well. Therefore, the general increase in dispersion in our study due to the stenosis might be explained by the noncentric velocity profile in the stenosis region. This is caused by the bifurcation and the angle of the constricted vessel with respect to the LMCA (cf. Figure 6). Graafen et al. did not have these effects in their study, since they were using a single straight vessel geometry. 

The lower dispersion inside the stenotic LAD branch for pulsatile flow for full autoregulation compared to the dispersion inside the normal LCX branch might be explained by the formation of an eddy behind the stenosis (cf. Figure 6). An eddy motion could be an evidence of turbulent flow. If turbulences occur, these could cause an increased radial exchange of contrast agent, which would lead to reduced dispersion in flow direction. For the healthy vessel, the variance of the VTF is increasing almost linearly behind the formation of a parabolic velocity profile, which was already shown by Graafen et al. [15].

#### 4.1.3. Comparison between Pulsatile and Constant Flow Simulations

Since computing time differs strongly for pulsatile and constant flow simulations, it was tested whether it is sufficient to consider a constant flow for CFD dispersion simulations. The results show that in the stenotic branch dispersion differs strongly for pulsatile and constant flows, being smaller for pulsatile flow (cf. Figure 7). A possible reason for this difference might be the displacement of the single laminar layers with respect to each other due to the impact of the pulsatile wave on barriers, in this case the stenosis. Because of this displacement, an increased gradient of contrast agent concentration arises perpendicular to the flow direction leading to an increased exchange of contrast agent between the laminar layers. This yields a reduction of dispersion in flow direction. The results show that simulations might be performed under constant velocity condition inside normal vessels for a rough estimation of dispersion, since almost no difference can be seen for pulsatile and constant flows in this normal branch. The advantage of this is that the computing time for constant flow is only about 5% of the computing time for pulsatile flow. However, negligence of pulsatility leads to an overestimation of dispersion inside stenotic vessels and should therefore be considered. 

#### 4.1.4. Bolus Dispersion for Full and Limited Autoregulation

The degree of autoregulation in the stenotic branch shows a strong effect on the contrast agent bolus dispersion. An increase in dispersion was found inside the stenotic branch for limited autoregulation compared to full autoregulation for both rest and hyperemia. The reason for this is the negative correlation between velocity and dispersion [15, 16]. Furthermore, no obvious eddies have been observed behind the stenosis region for limited autoregulation in contrast to full autoregulation. This eddy might lead to a smaller dispersion, which is described above. The two outflow conditions considered here for the constricted LAD represent two extreme situations that could arise for a stenosed vessel. Stenosis with a low degree might be compensated by the downstream microvascular network, (i.e., full autoregulated results). Segal et al. measured a reduced velocity proximal and distal to stenosis when measuring the time-averaged peak velocity before and after angioplasty [26]. The stenosis used in our simulations showed an area reduction of 80%. In contrast, the averaged area reduction inside the stenoses regarded in the study of Segal et al. was 96%. After angioplasty, the averaged area reduction was reduced to 55%. Therefore, the real outflow condition for a stenosis of an area reduction of 80% probably lies in between the two situations considered in our study. In general, the area reduction of the stenosis has to be considered to choose proper flow conditions through the stenosed vessel in future simulations since results for dispersion might differ significantly for unrealistic outflow conditions. 

### 4.2. The Influence of Bolus Dispersion on MBF and MPR Quantification

The degree of autoregulation has an important impact on the MBF and MPR errors as well. For full autoregulation, which can be expected for an early stenosis stage with low area reduction, the MPR inside the stenotic branch is overestimated with respect to the MPR inside the normal branch (cf. Figure 9). In contrast, for the case of limited autoregulation, the error in the MPR inside the stenotic branch is smaller compared to the results obtained for the normal branch. However, the early disease stage may be of particular interest to prevent the patient from negative disease progression. The more pronounced overestimation of the MPR in the tissue supplied by a stenotic vessel in comparison to the healthy tissue reduces their distinguishability. Unwanted false negative findings of the perfusion analysis may occur. As a consequence, realistic outflow boundary conditions are essential. A difference can be seen in the error of the MBF for pulsatile and constant flows in the stenotic LAD. Furthermore, the overestimation of the MPR is larger for pulsatile flow compared to constant flow for the stenotic branch. This fact affirms that pulsatile flow should not be neglected, especially for stenotic vessels. 

In general, the MPR error is relatively small compared to the interquartile range of about ±20% for myocardial perfusion MRI caused by measuring inaccuracy and inter-patient variability, which was observed in healthy volunteers [7]. But since the errors in the MBF values are significantly larger and not negligible, they might affect the diagnostic precision of CAD at myocardial MR-perfusion measurements. In contrast to former studies dealing with the error in MBF and MPR due to bolus dispersion with even more simplified coronary artery geometries [15, 16], a not negligible error in MBF up to −19.1% for constant flow and up to −16.1% for pulsatile flow was found in our study. This fact supports further CFD simulations with even more realistic geometries and boundary conditions to investigate the error in MBF and MPR more precisely. The difference in the error in MPR concerning pulsatile and constant flows is low, but not insignificant. Graafen et al. were exploring the difference in bolus dispersion due to variation in heart rate and systolic-to-diastolic duration ratio and found only small deviations of about 1-2% [16]. Therefore, we think that pulsatile flow should be considered in future simulations, but it might be sufficient to use a standard velocity pattern combined with a mean patient-specific velocity, instead of measuring an individual velocity pattern for each patient. In general, an overestimation of the MPR was found for each case. This overestimation could lead to a wrong classification of an ill patient as healthy, that is, a false negative finding.

### 4.3. Limitations

Main limitations of this study arise from simplifications concerning the coronary artery geometry, the boundary conditions, the laminar flow condition, and the rheological properties of blood and contrast agent. 

The geometry was build up out of straight cylindrical tubes. The real coronary tree includes a number of bifurcations, smaller branches, curvatures, and noncylindrical shape with changes in diameter along the vessel. Therefore, simulations in more realistic geometries of the coronary tree would be beneficial. Furthermore, the implementation of more realistic outlet boundary conditions, for example, via lumped parameter models [40, 41], might be interesting to consider in future studies. Wall motion due to pressure waves and general movement of the coronary arteries due to heart contraction and respiratory motion may as well influence contrast agent dispersion and should therefore be included in further simulations. The vessel wall was assumed to be rigid. We think this assumption is appropriate for a first investigation because of two reasons: (i) since coronary arteries are muscular arteries, their overall elasticity is small compared to larger elastic vessels, (ii) atherosclerosis, which is manifested in the occurrence of stenosis, leads to a reduced wall elasticity [42]. Several CFD studies have been investigating the influence of vessel wall elasticity on the velocity profile and wall shear stress, for example, [43–45]. Due to the consideration of special conditions and different vessel types and geometries in those studies, their results cannot be transferred directly to our model. Since Kabinejadian and Ghista found that the changes in the velocity pattern due to consideration of wall compliance are not significant [43], a substantial influence of the wall compliance on the dispersion of contrast agent is not expected. However, a difference can be seen in the axial velocity profiles of the rigid and compliant wall model [43]. Furthermore, less flow separation and reversed flows were observed regarding a compliant wall compared to a rigid wall [43], which might result in a smaller dispersion of contrast agent. Therefore, the effect of a compliant vessel wall on the dispersion of a contrast agent bolus should be inspected in future studies.

In general, depending on the geometry of the bifurcation, the flow division and the inflow conditions, secondary motion, and even flow separation at the outer wall can occur within the bifurcation area [38]. In case of multiple bifurcations inside the coronary tree this might result in a smaller dispersion than expected. Furthermore, the dispersion inside junctions of small vessels from a large vessel has to be investigated. Future studies regarding multiple realistically arranged bifurcations have to be performed to investigate the observations presented here more precisely.

The velocity pattern used for the simulations was scaled depending on the respective condition. In reality, velocity pattern shapes for hyperemia condition might look different than patterns for rest condition. Moreover, patterns measured distal to a stenosis were observed to have a more pronounced systolic component compared to a pattern measured inside healthy vessels [26]. These effects were neglected in this study as well.

In addition, we considered a laminar flow. However, close to the bifurcation and especially in the stenosis region, turbulences, secondary flow, and even flow separation might occur [46]. Due to the grid resolution only larger eddies are considered in our study. Reynolds numbers up to 1340 were found inside the stenosis center for pulsatile hyperemic flow and full autoregulation. These are well above the maximal value of *Re* = 750 for limited autoregulation. This difference might explain the appearance of an eddy in the region behind the stenosis for the case of full autoregulation and its absence for limited autoregulation (cf. Figure 6). The maximal Reynolds number for full autoregulation lies clearly below the critical value for flow inside a tube (Re_krit_ = 2300). However, the critical Reynolds number for turbulences clearly falls for stenosis with a diameter reduction of more than 25% [47]. If a turbulent flow is apparent, the resulting velocity profile is flattened compared to a fully developed laminar (i.e., parabolic) velocity profile due to energy exchange in radial direction. The dispersion of the contrast agent bolus is reduced in this case. Our simulations may serve as a worst-case estimation of laminar flow. However, the implementation of a turbulence model, for example, the *k*-*ω* model [46], in future simulations would be interesting.

The fact that rheological properties of the contrast agent were neglected seems to be acceptable since the mass fraction of contrast agent of the blood-contrast agent mixture is sufficiently small. One further point to consider could be the non-Newtonian behavior of blood, which is important to consider for small vessels with a diameter less than 1 mm [48]. Since the diameter of the vessels considered in our study is larger than this value, the assumption of a Newtonian fluid appears to be acceptable. However, several CFD studies regarding coronary artery geometries have been performed considering the non-Newtonian behavior of blood [10, 43, 49]. Chaichana et al. were comparing their results for wall shear stress for non-Newtonian and Newtonian models [49]. Results were found to be similar for both models, but more detailed for the non-Newtonian model. Kabinejadian and Ghista found a flattened axial velocity profile which was less skewed towards the outer wall of a curvature at regarding a non-Newtonian model [43]. This flattened profile might cause a smaller dispersion of contrast agent. Therefore, blood should be considered as a non-Newtonian fluid in future simulations. 

In general, patient-specific simulations would be the most realistic solution. Coronary artery characteristics are different for each individual person. Therefore, a realistic estimation of the bolus dispersion for an individual is only possible by accounting for individual geometries and boundary conditions. On the other hand, simulations performed in idealized geometries can help to improve the understanding of the bolus dispersion for different conditions step by step. The need for patient-specific simulations has to be examined in future studies.

In this study, the influence of bolus dispersion on the MBF and MPR determination with the tracer kinetic model MMID4 was analyzed. Another model used for quantitative estimation of the MBF is the Fermi function model [5, 50]. Furthermore, semiquantitative methods for the analysis of the data obtained during MR-perfusion measurements exist [3, 4] and are more common in clinical practice compared to quantitative analysis. The influence of the bolus dispersion on the MBF determination using these models could be applied and investigated in future studies.

## 5. Conclusions

The aim of this study was to examine the impact of contrast agent bolus dispersion on the quantification of the MBF and MPR considering a simplified coronary artery bifurcation. In the region behind the bifurcation, the increase in dispersion was slightly reduced. In most simulations, dispersion was larger in the stenotic branch compared to that in the normal branch. Furthermore, dispersion was less pronounced inside the stenotic branch for pulsatile flow compared to constant flow. For the limited autoregulation condition of the stenotic branch, the dispersion and error in the MBF is increased compared to results for the full autoregulation condition. A systematic underestimation of the MBF and an overestimation of the MPR were observed. An underestimation of the MBF up to −16.1% for pulsatile flow is not negligible compared to the interquartile range of about ±20% for myocardial MR-perfusion measurements in healthy volunteers [7]. Therefore, dispersion should not be neglected. The overestimation of the MPR might cause a false negative classification of a patient, especially in an early stage of CAD.

## Figures and Tables

**Table 1:** List of the outflow conditions, scaling factors for the original pulsatile velocity pattern, and associated mean velocity values that have been used for the constant velocity simulations.

		Outflow condition (LAD/LCX) (%)	Scaling factor pulsatile	Mean velocity (m/s)
Full autoregulation	Rest	50.0/50.0	1.000	0.200
	Hyperemia	50.0/50.0	2.300	0.460

Limited autoregulation	Rest	45.3/54.7	0.915	0.183
	Hyperemia	35.1/64.9	1.773	0.355
